# The theater and economy of nature: religion and the investigation of nature in seventeenth-century Dutch colonial Brazil

**DOI:** 10.1590/S0104-59702025000100020

**Published:** 2025-05-02

**Authors:** Christian Fausto Moraes dos Santos, Marlon Marcel Fiori

**Affiliations:** i Associate Professor, Universidade Estadual de Maringá. Maringá – Paraná – Brazil. chrfausto@gmail.com; ii Researcher, Universidade Estadual de Maringá. Maringá – Paraná – Brazil. marlonfiori@gmail.com

**Keywords:** Dutch Brazil, Economy of nature, Theater of nature, Seventeenth century, Colonial Brazil, Brasil holandês, Economia da natureza, Teatro da natureza, Século XVII, Brasil colonial

## Abstract

Historians tend to agree that the investigations by Piso and Marcgraf into nature in northeastern Brazil during the brief Dutch rule in that region were guided by a profound utilitarian approach. This article broadens this perspective by demonstrating how religious elements were heavily involved in their investigations of the natural world. We suggest that for both, like many of their contemporaries, the study of nature was also a way to contemplate the wisdom and omnipotence of the creator’s work and demonstrate how religious elements, like the idea of the economy of nature, influenced the understanding of the natural world.

## Resumo

Os historiadores normalmente convergem para a ideia de que as investigações de Piso e Marcgraf sobre a natureza no Nordeste do Brasil, empreendidas durante o breve domínio neerlandês nessa região, foram orientadas por uma profunda abordagem utilitarista. O artigo amplia essa perspectiva, demonstrando como elementos religiosos estiveram fortemente envolvidos na investigação deles do mundo natural. Sugerimos que, para eles, assim como muitos de seus contemporâneos, o estudo da natureza também era uma maneira de contemplar a sabedoria e onipotência da obra do Criador e demonstramos de que modo noções religiosas, como a ideia de economia da natureza, influenciaram a maneira pela qual o mundo natural era compreendido.

In the summer of 1636, the Dutch West India Company appointed the German Count Johan Maurits Nassau-Siegen (1604-1679) governor-general of the colony it maintained in northeastern Brazil, a post he held for seven years until May 1644. Nassau brought with him a group of scholars and artists responsible for studying and depicting the most varied aspects of Brazil (see Gesteira, 2022, p.43-46). Under the Count of Nassau’s patronage, sophisticated maps of the cities, local buildings and topography, as well as countless paintings and drawings which represented the views, inhabitants, fauna and flora of the colony were created.

The physician Willem Piso (1611-1678) and the astronomer and natural philosopher Georg Marcgraf (1610-ca.1644) were among the members of Nassau’s entourage. They both vigorously collected descriptions and information about the plants, animals, diseases, astronomy, geography, Indigenous people and other populations of Brazil, and brought these investigations together in two works: Marcgraf’s famous *Historia naturalis Brasiliae* [The natural history of Brazil] appeared in 1648, followed ten years later by Piso’s book entitled *De Indiae utriusque re naturali et medica* [On the natural and medical things of both Indies].

As a number of historians have noted, these investigations share a profound utilitarian approach to the natural world. Interest in the use of plants and animals was, of course, nothing new. In the Modern Era, attitudes about the usefulness of such creatures gradually changed; during the Renaissance, animals and plants could be beneficial to men in various ways, including in sermons or even as moral examples, but in the early Modern Era the use of plants and animals began to focus on material exploitation (Harrison, 2004). In the case of the Dutch colony in Brazil, the most detailed knowledge possible on geography, environment, diseases, fauna and flora was a way of ensuring control over the territory, generating material and symbolic profits and ultimately reinforcing the ideal of Empire, which characterized European expansion and colonization in the Modern Era (Asúa, French, 2005, p.232-233; Gesteira, 2006; Teixeira, 1995, p.91-92; Françozo, 2014).

Yet, this perspective obscures some crucial aspects of Piso and Marcgraf’s work. They shared a deep interest in investigating the natural world that extended beyond just material benefits or helping to expand knowledge about the cosmos. In the early Modern Era, the study of nature was also a path to devotion (Jorink, 2010) and nature also reflected God’s benevolence, the providence of a divine economist.

In analyzing the works of Piso and Marcgraf, this paper discusses how religious elements profoundly influenced the way they both studied and understood the natural world. On the one hand, we want to suggest that the study of nature was also a way for them to contemplate God the creator and his providence, a perspective that was also shared by many of their contemporaries. On the other hand, we also want to discuss to what extent religious notions (such as the “economy of nature”) were involved in how the natural world was understood, especially by Piso.

## The book or theater of nature

From the very first pages of *On the natural and medical things of both Indies*, Piso made it clear that the purpose of the book was neither to report sensationalist findings nor satisfy human egos. In fact, the objective was much more pious and fascinating: wrote that the book “does not intend to be an interpreter of things imagined for the delight of the senses and the lure of pleasures, or equipped with pomp and vast ostentation, but declares to deal with the works of the supreme creator in a simple style” Piso (1957, p.4).

This purpose reveals a widely held conviction in the early Modern Era, that the observation and study of the natural world made it possible to contemplate the omnipotence of God. Nature was considered a manifestation of divine power and greatness, thus offering two paths to knowing the divine: through the Bible and through God’s works in creation. This idea that creation is a second revelation of God essentially derives from two different sources: classical philosophy and especially church fathers and the Bible (Bright, 2008). Of course, the most important source for this doctrine was the scripture; several passages mention how God’s hand could be recognized in his works.^1^ Scripture and nature were sources of knowledge about God, and both share the same goal of declaring divine glory (Jorink, 2010; Howell, 2008; Koppenol, 2007, p.515).

The idea that nature could be compared with a second revelation of God was expressed by modern authors through a variety of metaphors: nature was a mirror, a work of art with divine authorship, a building, or more commonly a theater or book (Jorink, 2010, p.34-35; Findlen, 1996, p.55-57). This is clear in the *Belgic confession* (1561), one of the founding documents of the Dutch Reformed Church. Article II states that God “makes himself known to us more openly by his holy and divine word” and also “by the creation, preservation, and government of the universe, since that universe is before our eyes like a beautiful book in which all creatures, great and small, are as letters ‘to make us ponder the invisible things of God’*”* (Jorink, 2010, p.20; emphasis in the original).

The metaphors of theater and the book of nature frequently appear in *On the natural and medical things of both Indies*. Above all, the nature of the New World was a theater (Piso, 1957, p.8, 45, 556) that permitted contemplation of the wisdom and wonders of divine creation. In a letter to Johannes Antonides van der Linden (1609-1664), attached in an excerpt of the book, Piso (1957, p.556) emphasized that his work addressed “things of the extremely happy theatre of the old nature, observed in the New World.” In another passage, in a probable allusion to the parable of the talents,^2^ he wrote that with the support and encouragement of Nassau he had investigated the natural and medical history of Brazil, since “I should not hide this talent, which God has entrusted to me to sing his glory, thus avoiding becoming a useless servant” (Piso, 1957, p.4).

The conviction that nature was a source of knowledge about God shared a universal message (Bono, 2008, p.300; Jorink, 2010, p.36-37): the natural world was a theater, where all men could admire God’s omnipotent and benevolent works. It was a permanently open book, that could be consulted and read anywhere.^3^ God had manifested himself to Christians through the Bible, but the infidels could not deny having received the truths of faith, since nature was a book or theater accessible to all.

The vocabulary of God’s creation was intelligible to everyone everywhere, and the New World was obviously no exception. Piso highlighted how the signs of God’s omnipotence were evident in the majestic diversity of plants and other beings found on the other side of the Atlantic. As a part of God’s greatness, nature in the New World was an accessible (albeit neglected) path that could lead the natives to the “true God:”

It is worthy of observation that so many remarkable trees, shrubs, and herbs, with the exception of a few, in form, leaves, and fruit, appear different from the vegetables of the Old World. The same can be seen in birds, animals and fish, in winged and non-winged insects. … All these, with ineffable beauty of colors and in infinite multitude are produced in these regions, partly known to us, partly unknown. Some of them exceed European ones in taste and quality. Others are far inferior to them. From which it emerges how extraordinary and varied is the caprice of nature on the earthly globe. The innocent would deservedly be considered happy if they knew the creator of all these things (Piso, 1957, p.48).

Nature reflected a universal divine revelation, so the illiterate natives of the New World and infidels had no excuses for not recognizing the creator. God had expressed himself to men through the Bible as well as through the plants and each of the creatures in the firmament. Herbs, trees, animals, and minerals were sources of sacred knowledge, and the testimonies of divine wisdom hidden in the natural world seemed endless: they only needed to be piously observed.

## God’s goodness and providence in the creation

By the 1600s, serpents recurrently appeared in stories and texts about the fall from Eden and the doctrine of original sin. The Italian priest Giovanni Battista Hodierna (1597-1660), who wrote several works on astronomy and natural history, suggested that snakes used their forked tongues to clean dirt from their noses (Morgan, 2008, p.52), possibly alluding to the story in the book of Genesis where God condemned the serpent to forever crawl the earth swallowing dust as punishment for tempting Eve (The Hebrew Bible…, 1989, Gn 3, 13-14). Serpents were equally recurrent in emblem books, where they represented many qualities and meanings ranging from wisdom, wit, spiritual renewal and medical help to perfidy, poisoning and death (Daly, 2014).

Besides texts on religion and emblems, serpents frequently appeared in medical treatises and medical and surgical manuals. Physicians were very interested in their venomous bites that required treatment, and several Greco-Roman authorities devoted many pages to snakes and their venoms. At the same time, Europeans also had access to a wide tradition of Arabic texts on the topic: in the early Modern Era, Avicenna and Maimonides (whose works were translated during the Middle Ages) were some of the main references (Walker-Meikle, 2014).

Like many others, Piso was deeply intrigued by the venomous animals he found in Dutch colonial Brazil (principally snakes), their venoms, and the appropriate antidotes available for treating them. Piso reported that all evidence collected in Brazil indicated that snake venom was located in the head as well as certain viscera. He also observed that the natives kept snake heads for external application to human body parts that had been bitten, believing them to be effective against venom. In Piso’s view, this treatment (and its proven efficacy) was a clear example of God’s greatness and wisdom in creation which was manifested even in these animals condemned to drag themselves on the ground:

It is easily deduced that the venom of snakes is hidden in the head, because they serve as food for the snake-eating inhabitants, amputating their heads; the opposite happens to them, when they taste the smallest part of the head or certain viscera. [The natives] usually keep the heads for external remedies against snake bites ... As if there were no poison so atrocious, created by God, that it did not provide liberal aid in medicine, and the reptiles themselves, in their perpetual crawling, magnified the majesty of the creator (Piso, 1957, p.570).

All the characters in the book of nature thus seemed to manifest God’s goodness, and even venomous animals and poisons reflected something of God’s providence. But these wonders were visible not only in serpents; investigation of the oceans, according to Piso, was also an endless source of admiration for God’s almightiness. “Since the admirable power and ability of God are revealed to all in heavenly things and in what is operated in the air and on earth” he wrote, reiterating the underlying universality of divine creation, “it seems, however, much more to be displayed at sea, where so many and stupendous forms of things are seen and their strengths and properties are revealed, so that one never stops researching and contemplating, and with good reason it can be said that the known part is a tiny fraction of the unknown” (Piso, 1957, p.47).

As in the seas and even in the creeping and venomous reptiles, divine wisdom also was visible even in the most minute creatures. Insects, these small and innumerable beings with so many unusual shapes and behaviors, revealed the hidden work of the divine, providing even more proof of God’s hand in creation.

From around 1570, insects increasingly fascinated Europeans and circulated more frequently and more prominently in books, artworks and cabinets of curiosities (Neri, 2011; Ogilvie, 2008; Jorink, 2010, p.181-252; Jorink, 2018, p.131-147). In 1602, Ulisses Aldrovandi’s (1522-1605) *De animalibus insectis libre septem* [Seven books on the insect animals] was published, and was followed almost thirty years later by Thomas Mouffet’s (1553-1604) *Insectorum sive minimorum animalium theatrum* [Insects, or the theater of smallest animals]. But most of the material for printing both texts was gathered in the 1580s and 1590s, and although Mouffet’s work was not printed until 1634, three decades after his death, the manuscript was completed in 1598.^4^ The attention that Marcgraf and Piso devoted to insects is indicative of this new attitude: their observations and descriptions involve a diverse group of species extending beyond ants, bees and butterflies to flies and mosquitoes, beetles, caterpillars, spiders, scorpions, cicadas, chiggers, ticks and numerous others. All in all, book VII of *The natural history of Brazil*, entitled *De insectis* [On the insects], contains descriptions of roughly fifty of these animals, interspersed with various illustrations.

Although the classical authors did not dedicate any work exclusively to insects, several ancient texts contain information and mentions of these creatures. Insects are commonly cited as metaphors in poems, satires, dramas, fables, tragedies and comedies, seen as sources of moral teachings or models and paradigms of human virtues and vices. Ants, bees and wasps were frequently mentioned, and characteristics including their complex social organization, caste systems, building skills, dedication to work and cooperativeness were often correlated with human behavior (Egan, 2014). In the early Modern Era, however, scholars often turned to what Pliny and Aristotle had written as some of their main sources of information about insects.

Aristotle stated that insects lacked complex anatomy like that observed in higher animals, were completely devoid of blood (or, at least, red blood), and many were born from dung, dew on leaves, meat and decaying substances (Egan, 2014). In the encyclopedic *Naturalis historia*, all of book XI was devoted to *insecta*. Pliny addressed many of these creatures in his work, and some of his descriptions are extensive and detailed. Like Aristotle, he was convinced that many reproduced by spontaneous generation, but this did not mean they were to be judged disparagingly: he stated that “in the contemplation of nature nothing can be considered superfluous” (Pliny, 2003, p.458). Although he deemed elephants, lions and bulls were worthy of admiration, it was in the small creatures that the perfection of nature could be observed in its fullness (p.458).

The Bible likewise refers to these small creatures apparently devoid of blood, sometimes as a manifestation of divine wrath and sometimes as evidence of divine wisdom. Several passages present insects as devastating plagues that tormented men: Exodus and the Psalms (The Hebrew Bible…, 1989, Ps 105, 26-35; 78, 45-46; Ex 8, 15-22) mention God’s punishment of the Egyptians with clouds of gnats as well as hordes of locusts that devoured every herb of the land and the fruit of the field. In Deuteronomy (The Hebrew Bible…, 1989, Dt 28, 38-30), locusts are also listed among the punishments for the Israelites for disobeying God. In contrast, other biblical passages suggest how the wisdom and goodness of the creator can be seen even in the smallest of creatures, and emphasize how insects have lessons to teach men:^5^

Go to the ant, you sluggard; consider its ways and be wise!

It has no commander, no overseer or ruler, yet it stores its provisions in summer and gathers its food at harvest.

How long will you lie there, you sluggard?

When will you get up from your sleep? (The Hebrew Bible…, 1989, Prv 6, 6-9).

The legacy of classical and biblical sources shaped European concepts of the world of insects. When he returned to the Netherlands, Nassau donated part of the natural history collection he assembled in Dutch colonial Brazil to the nobleman Frederick William (1620-1688), Elector of Brandenburg.^6^ This gift included paintings by Albert Eckhout (1610-1665), furniture carved in ivory, and many oil paintings, watercolors and crayon/pencil sketches, especially portrayals of Brazil’s fauna and flora and Indigenous and African peoples. Around 1660, part of these illustrations were organized into four folio volumes by Christian Mentzel, the Elector’s private physician, under the collective title *Theatrum rerum naturalium Brasiliae* [Theater of natural things of Brazil].

Volume I of the *Theatrum* contained illustrations of fish, volume II included images of birds, and volume IV was dedicated to plants. Volume III was devoted to Indigenous and African peoples, quadrupeds, reptiles and also insects. Mentzel (1995, p.4), however, stressed that “as there was room in this volume, I decided to add the insects. They are imperfect animals that all the classics say are born from putrefying things.”

Marcgraf and Piso gathered much information about the insects they found in Dutch Brazil, which was supported by the knowledge of local Indigenous people and first-hand observations, but their descriptions also have a strong textual orientation, supported by biblical and classical sources. Symbolic meanings, for example, are evident in some descriptions: cunning spiders, industrious ants, the ingenuity and organization of bees ruled by a “king, whose body volume was thinner than the others and very golden” (Piso, 1957, p.258), or even praying mantises, which because of their slender appearance seemed to be omens of hunger or taught “men to raise their supplicating hands to heaven” (p.654-655).

Since some insects found in the colony were useful as medicine (like ants) or harmful to humans, it is not surprising that medical concerns also caught the attention of Piso and Marcgraf. Many of these creatures could pose health risks, so knowing how to treat their bites was important knowledge, especially for Dutch outsiders in a landscape that was still mostly unfamiliar. In the case of mosquitoes, for example, Piso wrote that they could be repelled with bonfires, candles and torches, or by anointing uncovered parts of the body with oils from native sources like *copaiba*, the Brazilian diesel tree. If bitten, victims were to refrain from scratching or using cold water on the affected parts, but instead resort to some herbal medicine of “cold property” (Piso, 1957, p.600-601).

Nonetheless, Marcgraf and Piso’s interest in insects extended beyond symbolism or medicine: their descriptions contained observations about the behavior of these animals, some quite curious. Marcgraf (1942, p.257), for example, wrote that cicadas burst from their giddy and prolonged chirping, as evidenced by empty shells with broken backs often found on trees. His descriptions also included numerous observations on reproduction, metamorphosis and especially the morphology of these creatures.

Even if Piso and Marcgraf shared the ancient idea that insects generated spontaneously, metamorphosis also indicated something prodigious. How could it not be surprising that certain caterpillars that devour cabbages in the garden turn into some of Brazil’s most exuberant birds, the hummingbirds? “When the aforementioned caterpillars begin to transform into these little birds,” noted Piso (1957, p.660), “first of all, the beautiful feathers with wings appear, so that the shape of the caterpillar can be clearly seen, as for the lower part of the body, already transmuted into another upper part into a bird [hummingbird].” These marvels arousing curiosity and admiration seemed to underscore the almighty nature of God in every detail of creation, even though what the physician believed an extraordinary feat was, in fact, the metamorphosis of the hummingbird hawk-moth from a caterpillar into a butterfly (see Daston, Park, 1998, p.311-316). God’s hand could be also recognized in the metamorphosis of locusts that became plants, as well as numerous other examples that, according to Piso (1957, p.655-656), dated back to the classical tradition and were reported by many of his contemporaries.

The morphology of insects was also wondrous: a close look at insects, whether these were beetles, caterpillars or mosquitoes, revealed the details and complexity of their external appearance. Marcgraf (1942, p.256) described the *jacatinga*, a species of dragonfly, detailing a bifurcated upper jaw, two upper teeth furnished with four stingers and two lower teeth with only one, hairy legs, thin nails, and membranous wings interspersed with tiny veins. Another of these tiny animals had bulging round eyes, teeth-like pincers, two small “horns” and a body covered with silk-like hair (p.256). Insect after insect, a series of details were carefully described.

The careful attention paid to insect morphology appears not only in the descriptions, but also in the images in *The natural history of Brazil*. Approximately thirty illustrations call readers’ attention to the diversity of forms, in many cases stressing the intricate and delicate appearance of external morphology. Even more impressive were the images of insects contained in the watercolor drawings known as *Handbooks*, *Manuais* or the *Libri Principis* which were also part of the volumes in which Mentzel assembled the collection of Brazilian nature art that Nassau donated to the Elector of Brandenburg.^7^ The authorship of many of these images is attributed to Marcgraf himself (Brienen, 2001).

Many of the insect descriptions without images in *The natural history of Brazil* have corresponding images that can be identified in the *Handbooks*, including the aforementioned *jacatinga*. Together, the two volumes of *Handbooks* contain seventy figures of insects, more than the total number of images of quadruped animals. Most of these images stand out for their realism and richness of detail. The many handwritten annotations in the *Handbooks* from Nassau reveal that these insects were painted to scale. The insects move from the margins to the center of the pages; most are depicted alone, but some appear in a set of three or four specimens. This vast group of images in the *Handbooks* constitutes one of the first (if not indeed the first) comprehensive surveys of insect depictions by Europeans in the New World.

Marcgraf and Piso had a valuable device: a magnifying glass. This allowed them to more deeply explore the intriguing world of insects, although as Marcgraf (1942, p.247) himself noted, this instrument only permitted magnification to a small extent. Unlike telescopes that required at least two lenses (one concave and one convex), a single lens could be used in a simple microscope. Such lenses were nothing new at that time, and were already used as spectacles and instruments for miniaturists, for instance. However, around 1620 they began to be used systematically for studying plants and insects (Lüthy, 1996; Ilardi, 1976; Jorink, 2010, p.209-219).

Microscopes and magnifying glasses made it possible to examine details that had previously gone unnoticed, providing an opportunity to access a new world of small creatures which had not yet been extensively explored. Marcgraf and Piso were fully aware of these advantages, and vigorously investigated these animals through their magnifying glasses. They were impressed with the tiny beings that could be revealed with a lens, beings that would otherwise go virtually or completely unnoticed. The *tunga*, a species of flea also known as the jigger or sand flea,^8^ Piso wrote, was “small and, hiding inside [the soles of the feet and even the palms of the hands], a little more deeply, enclosed in a round vesicle, they are shown in black, and it is convenient to examine them through the megascope; otherwise, not even by a lynx can [they] be rightly perceived” (Piso, 1957, p.601).

Piso (1957, p.601-602) observed that these insects reproduced on the heels, between the fingers, and on the soles of the feet and even hands of their hosts, noting that their offspring, when “crushed by the nails, crack like nits.” The curious *tunga*, however, was not the only small animal that could be inspected in more detail with a lens. Marcgraf (1942, p.259) described another creature that “would be almost imperceptible because of its smallness, if it weren’t black, glossy and entirely round.” He stressed that “with the magnifying glass, it appears to be the size of a hemp seed and a round, black, glossy figure. This insect is covered by a round shell and walks on six little feet.”

Magnifying glasses not only made it possible to recognize creatures that had been invisible or remained almost imperceptible to the eyes of European scholars, but also permitted careful examination of their complex morphology. With magnification, insects were full of unexpected structures and details. On the head of a certain species of mosquito, Marcgraf (1942, p.253) noted, “there are two horns, which can only be observed through a magnifying glass.” Another small creature exhibited the “figure of a millipede, when observed through a magnifying glass,” endowed with “six legs” and “two horns” (p.259). Equally intriguing were fireflies: through a lens, wrote the astronomer and natural philosopher in another passage, “I observed two hairs of beard, each one composed of fourteen particles; it is also observed that each foot has four toes, the legs are pointed and hairy … and, close to the mouth, there are four pincers” (p.258).

When the innumerable ants of the New World were observed more closely, variations in their coloring, dimensions and external characteristics became more evident. Ants in a colony, noted Piso (1957, p.605), “differ in size and color, but, well examined by the megascope, they also differ among themselves in the conformation of the parts.” As in the case of ants, a closer look at beetles also revealed something extraordinary: a species found in the colony and of “rare conformation,” Marcgraf (1942, p.247) noted, had a cavity in the front part of the body, where “you can see a large number of live embryos, dark in color, with some filaments, with which attach to the cavity.” “These ferns,” added the astronomer and natural philosopher, “are the size of a poppy seed, but with a magnifying glass, each one equals the size of a pea grain, similar in everything to the horned father.” Marcgraf must have been impressed. But what he considered to indicate something akin to a beetle’s dutiful parental care for its offspring today entomologists would interpret as mites (which can infest some coleopterans in large numbers) or pseudoscorpions (which are also found in the larger species).

The use of optical tools opened a new perspective on how Europeans could investigate the world of insects. Magnifying glasses and microscopes allowed researchers to observe insects as they had never been seen before. At the same time, these tools had a huge impact on the way these animals were represented. Books began to display images of tiny and strange creatures, enlarged and with an unprecedented degree of detail (Meli, 2010; Neri, 2011). Tiny insects were illustrated on the scale of mice. “The reader should know,” Piso (1957, p.599) proudly mentioned, “that this engraving of the *Nhatiu* [mosquito], along with those of the other tiny insects, is six times larger than the living animalcule, and for that reason it was drawn using the megascope, without whose help it was impossible to know its very fine conformations and articulations.” In the 1660s, as lenses and microscopy techniques continued to improve, such images of insects became even more impressively accurate (Jorink, 2010, p.219-239).

The growing interest in insects was stimulated to a large extent by the conviction that all kinds of living beings manifested the glory of God, and tiny insects were no exception. “Even in the smallest things, in fact, the power of the author of nature is evident,” wrote Piso (1957, p.660). In the book of nature, even the smallest characters demonstrated divine providence; it was in the minuscule structure of insects, their intricate reproduction and metamorphosis, that the creator’s genius was revealed. In *On the natural and medical things of both Indies*, Piso (1957, p.557) wrote that one of his purposes was to investigate “the nature and metamorphosis of some insects, truly admirable, great testimony that leads us to eternally celebrate the power and wisdom of the creator.”

In this way, during the seventeenth century, many theologians, physicians and scholars shared the idea that studying insects was a way to contemplate the might of God. The creator’s wisdom and providence could be seen not only in huge elephants, but also in flies, worms and ants. The delicate and complex structure of the insects was evidence of God’s constant care and presence in creation, and they were one of the best places to recognize the hand of God. As Piso (1957, p.258) declared, after Pliny, perhaps “nature never wanted to appear more perfect in anything than in the smallest things.”

## The economy of nature

In the theater or book of nature, every creature (even the smallest) provided traces of God’s wisdom. But such signs did not stop there: nature as a whole seemed to manifest divine providence, an economy of God. The boundaries of this idea of an economy of nature were fluid (Remien, 2012), but in general, the natural world was considered to be diligently and benevolently arranged by its creator. In this way, the economy of nature depicted God’s amazing ability to manage the cosmos, so natural resources were equitably distributed, so that each part regulated itself and performed its function efficiently. Nature reflected a divine economy, ensuring the order and maintenance of things, supplying enough but not too much.^9^ In *On the natural and medical things of both Indies*, Piso explicitly alluded to the notion of the economy of nature, which to the four corners of the globe manifested the organization of an industrious economist:

It seems to me that you agree that there are certain things mainly in certain places, and others do not appear there; and this with such a regimentation of nature that these beings appear superior or inferior, both in terms of number and mode of life and longevity. This truth is confirmed, at every step, by all the intrepid travelers of the Indies, which is also very consistent with the economy of Nature, that no place is given a luxuriant abundance, or the scarcity of necessary things reigns (Piso, 1957, p.227).

The term *oeconomiae* is derived from the Greek *oikos*, used to refer to the household and law. This term was already present in a variety of contexts in the early Modern Era, but around 1650 it was increasingly used by theologians, physicists, philosophers and natural historians to refer to God’s management and administration of the natural world (Schabas, Marchi, 2003). Many premises of the economy of nature were supported by conceptions of God’s design and providence, which sought to understand beneficent devices in living beings and hidden divine purposes behind the characteristics of the natural world. Johannes de Laet, who served as director of the West Indie Company and was responsible for editing *The natural history of Brazil*, wrote in his *New World or description of the West Indies* (1625) about how in certain provinces of Guatemala a certain species of “deer” was found, “to whom the Author in nature gave two ventricles, one to digest his food, and the other, as has been observed, to put rotten wood, without knowing for what use, although it is to be believed that nature does nothing in vain” (Laet, 1988, p.519). In another section of this work, he pointed out that on the bountiful and healthy island of Jamaica, there were “fruit trees which the Author of nature planted from the beginning by his benevolence” (p.100).

In the economy of nature, the natural world was understood as a judiciously and efficiently managed larder, and the abundance, variety, and distribution of created things were proofs of God’s omnipotence and benevolence. Some vestiges of this economy seemed evident. For example, how could God deprive some men of the gift of bread? But instead of wheat, New World natives could rely on nutritious cassava tubers. Considered the “main food of America” (Piso, 1957, p.55), Piso pointed out that “until this time many regions of the Indies lacked wheat; however, the benign Mother Nature did not want those things that sustain the life of men and brutes and that supply the wheat. Because the root cultivated or treated by the barbarians called *Mandihoca* [*sic*], reduced to flour and cooked like bread, competes with the best bread made from flour” (p.261).

The predominance of man over other animals was exalted by a variety of authors in the Modern Era. God created nature for man’s use and enjoyment, so that other species could be subordinated to human needs (Thomas, 1989, p.21-42). The physician John Rowland (1658, p.A3 verso) wrote that although animals could be a divine instrument for chastising sinners, God had selected man above all other beings and benevolently bestowed upon him all creatures for “for food, and raiment, and other necessary uses; also for his pleasure and recreation.” This dominion of humanity over the natural world could easily be justified through a literal interpretation of the Bible. Genesis describes how God blessed the first man and woman, saying: “Be fruitful, and multiply, and replenish the earth, and subdue it: and have dominion over the fish of the sea, and over the fowl of the air, and over every living thing that move upon the earth” (The Hebrew Bible…, 1989, Gn 1, 26-28). Classical authorities also provided similar arguments (Thomas, 1989, p.30).

Piso (1957, p.563) addressed the same correlation between use, God and the natural world. “What is there, by immortal God,” he asked, “of which no use is derived?” All created things seemed to correspond to a divine useful purpose. Even harmful things or beings like venomous animals and poisonous plants were seen as manifestations of God’s providence. “From the scorpion, the scolopendra, the snake and other serpents,” Piso wrote, “the bite is cured with their own medicine.” From the roots of cassava, “which has a lot of poison, you get great food and also a counterpoison.” He concluded that “one should not think that benign Nature would have denied a remedy when it created the disease, but to every poison it added and opposed an antidote” (p.563).

So, according to Piso, even poisonous plants and animals were somehow useful to men. This same conviction was shared by many other scholars in the Modern Era, although arguments for how poisons, beasts and noxious plants could be of use varied considerably. The renowned German polymath Athanasius Kircher (1602-1680) wrote that poisonous plants, minerals and animals were proof of God’s benevolence, since they served to purify the earth and air, making them suitable for human inhabitation (Baldwin, 1995, p.400). Piso’s vision, however, was not strictly anthropocentric; although it seemed consistent that for every poison there was an antidote in creation, not all toxic substances or harmful things seemed ultimately useful to people. Indeed, harmful poisons, plants, animals and even minerals seemed to fit into the economy of nature. “Although the entire earthly orb has a large quantity of poisons, which animals, plants and minerals are full of everywhere,” Piso (1957, p.562) wrote, “it still happens outside the primordial purpose of nature to produce harm and damage for someone. What for many is food, for others is poison.”

In other words, some things were harmful and of little use to men, but they had their place in God’s economy. According to Piso (1957, p.562), numerous examples could be easily listed, stating “The sea hare is harmless food for the large-scaled gurnard, and poison for us; and, in turn, what for one is poison, such as hellebore and hemlock for man, for some animals, such as goat, starling and quail, serves as food.” Moreover, Piso (1957, p.563) believed that the instinct of men and animals seemed to be in accord with God’s plan to secure the order of nature’s economy. The “rational and irrational animal was given an instinct,” he pointed out, “by which to wisely use all created things, choose the goods and utilities of life, and flee harmful things.”

The economy of nature to a certain extent shifted into another fundamental problem: the use of exotic *materia medica*.^10^ Were drugs from distant lands suitable for Europeans? Should Europeans take advantage of remedies found in the New World and the East, or were native remedies healthier? These questions were not new, dating back to Pliny’s time, but the debates became even more accentuated in the Modern Era, mainly as expanding streams of *materia medica* arrived in Europe from all over the globe (see Cooper, 2007; Wear, 1999; Spary, 2003).

The notion that the environment was intimately correlated with people’s physical characteristics, health and illnesses had been clearly expressed in Hippocratic medicine, notably derived from Greek notions of medicine and philosophy found in the texts of the *Corpus Hippocraticum*, mostly written in the late sixth and early fifth centuries before the Christian era. Hippocratic medicine understood the body to be composed of four vital fluids or humors (blood, phlegm, yellow bile and black bile), each responsible for an essential function for survival. Perfect health depended on balancing the four body fluids, while illness resulted from disturbance of the humoral balance: the lack, excess, corruption, obstruction or displacement of any humor could cause serious illness and seriously endanger life.^11^ According to some works in the Hippocratic corpus, geography was deeply linked to local diseases and the health of men (Corpus…, 2005, p.94-129). In this way, considering that climate and location directly influenced the human body, it was reasonable to assume that local remedies would not have the same effect on immigrants, or would not be suitable for treating illnesses from distant lands: native remedies provided the cure for local ailments.

Arguments in favor of native remedies were not only based on medical traditions, however. European greed for *materia medica* from distant lands seemed to run counter to divine benevolence and the economy of the natural world. Nature was welcoming, not neglectful. To no creature “nature wanted to be a stepmother, to the point of not supplying it with food and medicine in abundance, teaching also to distinguish them exactly from poisons,” stressed Piso (1957, p.55). The fact that the colony’s native trees in the scorching tropical climate bore mostly “cold” fruit seemed to reflect God’s economy in providing creatures with everything necessary in every corner of the cosmos. The wild trees of Brazil, Piso (1957, p.41) wrote, “bear beautiful fruits with a very pleasant taste, mostly cold and with an astringent property. As if the benign nature gave this comfort to mortals excessively punished by the heat and imposed a brake on the humors that are released.”

In Piso’s view, Europeans had no reason to prefer exotic remedies over those that grew and could be easily collected in their homeland. As an efficient economist, God had supplied the nations of the earth with plants and herbs suited for curing local diseases. Furthermore, the principles of Hippocratic medicine seemed to reinforce this same conviction:

In truth, so far am I from supposing that spices imported from the Indies, or medicines received from foreign peoples, should be preferred to those native to our country, that rather I believe that everywhere they have greater accord with human nature and are more efficacious in it those remedies which, because of the common ground and skies, are familiar to them; therefore it is more indecorous to ignore these than those (Piso, 1957, p.7).

We can assume that this conviction deeply irritated many of the directors and shareholders of the West India Company and especially the East India Company, which profited heavily from cargoes of Asian spices and *materia medica*. But in line with a classical Roman authority, Piso (1957, p.7) recalled God’s plan in distributing the components of the natural world. “To no one,” he wrote, “nature showed itself such a stepmother that it did not provide enough medicine and food.” The assimilation of Indigenous knowledge about local drugs was therefore fundamental to cure the Europeans in the colony, even if Piso believed that all this exotic *materia medica* was not suited to treat the illnesses of the Old World. The superiority of European medical knowledge over native New World medical theories, however, was beyond question.

Just as the idea of the economy of nature largely seemed to justify that the pharmacopeia available in distant lands was not suitable for Europeans, theological arguments were also raised by apologists for *materia medica* and other exotic products (Wear, 1999). In 1701, the abbot Pierre Le Lorrain de Vallemont (1649-1721) declared God had willed that “there should be a social bond between all men,” which is why “each country has its own gifts and advantages, and which trade and navigation make common to the regions they lack” (Vallemont, 1703, p.18-19). In his *Cosmographie* (1657), the English clergyman Peter Heylyn (1599-1662) observed that “God made the world and fitted it with all things necessary for the life of man, leaving man to provide himself of such Additions, as rather serve for comfort and conveniences in the way of his living” (Heylyn, 1657, p.4). He added:

But nothing more sets forth the power and wisdom of Almighty God, as it relates to these particulars, than that most admirable intermixture of want with plenty, whereby he hath united all the parts of the world in a continual traffic and commerce with another; some countries being destitute of those commodities, with which other abound; and being plentiful in those, which the others want (p.4).

In his *History of deeds recently practiced during eight years in Brazil and in other parts under the government of the illustrious Johan Maurits, Prince of Nassau* (1647), a voluminous overview of Nassau’s administration and generally a paean for the count’s achievements in Dutch Brazil, the renowned humanist and literati Gaspar Barlaeus (1584-1648) expressed a similar idea. In the first chapter, he stated:

We load our ships annually with these products [from the East Indies] and transport them to lands to which the creator of all nature has denied those seasoners of the cold of our climates. In this, the wisdom of God is admired, who wanted hot drugs to be grown in torrid regions, and cold ones in frigid regions, undoubtedly so that, by exchanging the products necessary for men, peoples would come together, forced by common necessity to become friends (Barlaeus, 1940, p.8).

Despite these discussions, the arguments of Piso and some of his contemporaries had no effect: increasing quantities of exotic medicines were exported to the Old World. Nevertheless, debates about exotic and native remedies and conceptions about the economy of nature indicate how various religious elements were deeply rooted in the study and interpretation of the natural world.

## Final considerations

Scholars have traditionally attributed the works of Marcgraf and Piso with a strong emphasis on the human utility of objects that in the early Modern Era reflected the notion of Empire, characterizing large-scale exploration and settlement by Europeans. However, Piso and Marcgraf’s works also point in another direction, demonstrating how religious considerations influenced or motivated study of the natural world and how many theologians, physicians, historians and natural philosophers understood nature.

For these authors, the study of nature was not just a route to discover natural products or *materia medica* that could yield wealth or be useful to men in some way. Nature was perceived like a theater, a book, and investigating it was a form of devotion, a way of admiring God’s might and wisdom in creation. In this vast theater of nature, all animals (even creeping and venomous animals like serpents) reflected divine wisdom. This was also true for insects, the most minuscule creatures, which seemed even greater proof of divine omnipotence and influence in all details of creation.

In Piso’s view, nature likewise reflected God’s benevolence and order in the cosmos, an economy of God, where resources seemed equitably distributed and all things properly adjusted, important and useful in some way. Interestingly, while Piso considered this economy of nature proof that God had provided all nations with everything they needed (including medicines needed to treat local ailments), many of his contemporaries tended to view it as a way of legitimizing exchange between men.

## Data Availability

Not deposited in a data repository.

## NOTES

1 Psalm 104, 24-25 (The Hebrew Bible…, 1989), for example, is entirely devoted to God’s majesty in creation: “How many are your works, Lord! In wisdom you made them all; the earth is full of your creatures.” Other passages such as Psalm 19, 1 contain similar messages.

2 One interpretation of the parable of the talents, which appears in the Gospels of Matthew and Luke, is that each person is entrusted with a certain occupation or talent so that when Jesus returns, everyone will be judged according to the acts they undertook with the talent they received (Kissinger, 1979, p.55).

3 See also the discussions in Gesteira (2004); Leite (2014); Kury, Camenietzki (1997).

4 On both works, see Neri (2011, p.27-74).

5 See, for example, Proverbs, 30, 24-28 (The Hebrew Bible…, 1989).

6 About the donations (gifts) made by Nassau to various individuals, see Brienen (2010, p.140-141); Whitehead, Boeseman (1989, p.25, 65-66).

7 On the *Libri Principis*, see Whitehead, Boeseman (1989, p.40-41); Brienen (2010, p.45-46).

8
*Tunga penetrans*, perhaps the smallest known flea species, measuring one millimeter (Linardi, 2005, p.402-403).

9 For a comprehensive discussion, see also Remien (2012); La Vergata (1988); Spary (2003).

10 The term *materia medica* collectively refers to medical specimens or parts of plants, animals and minerals employed in medicine in the early Modern Era. This idea will later be referred to as drugs or remedies.

11 On Hippocratic theory, see Edler (2006, p.34-37); Lima (1996, p.46-50).
